# The correlation between CYP4F2 variants and chronic obstructive pulmonary disease risk in Hainan Han population

**DOI:** 10.1186/s12931-020-01348-6

**Published:** 2020-04-15

**Authors:** Yipeng Ding, Yixiu Yang, Quanni Li, Qiong Feng, Dongchuan Xu, Cibing Wu, Jie Zhao, Xiaoli Zhou, Huan Niu, Ping He, Jianfang Liu, Hongxia Yao

**Affiliations:** 1 Department of General Practice, Hainan General Hospital, Haikou, 570102 Hainan China; 2 Hainan Affiliated Hospital of Hainan Medical University, #19, Xiuhua Road, Xiuying District, Haikou, 570102 Hainan China; 3 Hainan General Hospital, University of South China, Haikou, 570102 Hainan China

**Keywords:** Chronic obstructive pulmonary disease, Susceptibility, Agena MassARRAY technology, Case-control study, CYP4F2, Single nucleotide polymorphism

## Abstract

**Background:**

Chronic obstructive pulmonary disease (COPD) is a complex pulmonary disease. Cytochrome P450 family 4 subfamily F member 2 (CYP4F2) belongs to cytochrome P450 superfamily of enzymes responsible for metabolism, its single nucleotide polymorphisms (SNPs) were reported to be involved in metabolism in the development of many diseases. The study aimed to assess the relation between CYP4F2 SNPs and COPD risk in the Hainan Han population.

**Method:**

We genotyped five SNPs in CYP4F2 in 313 cases and 508 controls by Agena MassARRAY assay. The association between CYP4F2 SNPs and COPD risk were assessed by χ^2^ test and genetic models. Besides, logistic regression analysis was introduced into the calculation for odds ratio (OR) and 95% confidence intervals (CIs).

**Results:**

Allele model analysis indicated that rs3093203 A was significantly correlated with an increased risk of COPD. Also, rs3093193 G and rs3093110 G were associated with a reduced COPD risk. In the genetic models, we found that rs3093203 was related to an increased COPD risk, while rs3093193 and rs3093110 were related to a reduced risk of COPD. After gender stratification, rs3093203, rs3093193 and rs3093110 showed the association with COPD risk in males. With smoking stratification, rs3093144 was significantly associated with an increased risk of COPD in smokers. CYP4F2 SNPs were significantly associated with COPD risk.

**Conclusions:**

Our findings illustrated potential associations between CYP4F2 polymorphisms and COPD risk. However, large-scale and well-designed studies are needed to determine conclusively the association between the CYP4F2 SNPs and COPD risk.

## Background

Chronic obstructive pulmonary disease (COPD) is a commonly heterogeneous diseases caused by distinct pathophysiological processes, with a high morbidity and mortality [1, 2]. It is defined as an incomplete reversible airflow obstruction with persistent symptoms including dyspnea, cough, and excessive sputum production. As report went, it is the fourth leading cause of death (126, 000 deaths per year) [3], and accounts for 6.4% of the United States population self-reporting a diagnosis annually [4]. Despite the gradual improvement of people’s health awareness and detection technology of COPD, most patients with COPD have never been diagnosed up to 29 million people [5]. In the United States, 75% of COPD cases are diagnosed as smoking-related, but, other occupational or environmental factors such as diesel exhaust and smoke from indoor cooking contributing to the development of COPD [6]. In China, COPD was considered as the third leading cause of death and accounted for over 0·9 million deaths reported in 2013 [7]. And the latest Chinese national survey of COPD from 2002 to 2004 was conducted among 20,245 adults. The overall prevalence rate was reported up to 8.2%, thereinto, 12·4% in men and 5·1% in women [8], which was likely to associated with cigarette smoking in men [9]. Cigarette smoking is considered as the major environmental risk factor for the development of COPD. But individuals varied greatly in their susceptibility to response to tobacco smoking, illustrating that genetic factors played vital role in the incidence and development of COPD. Recent Genome-wide association studies have provided strong evidence for common susceptibility loci for COPD [10–13].

And Cytochrome P450 family 4 subfamily F member 2 (CYP4F2) encodes a member of the cytochrome P450 superfamily of enzymes involved in many metabolic pathways [14, 15]. It is responsible for metabolizing arachidonic acid to 20-hydroxyeicosatetraenoic acid (20-HETE) and involved in many reactions, such as drug metabolism [16], long-chain fatty acids metabolism [17], and synthesis of cholesterol, steroids and other lipids. Recently, Wang et al. elucidated that the upregulated differentially expressed genes were significantly enriched in the arachidonic acid metabolism pathway, including CYP4F2, PTGDS and PLA2G16 by pathway enrichment analysis and pathway interactive network construction [18]. Again, it’s involved in metabolic pathways.

Beyond that, CYP4F2 variants were involved in the development of some diseases.

Polymorphisms of CYP4F2 was reported to be linked with the susceptibility to cardiovascular and cerebrovascular diseases [17]. And variants related-Ischemic stroke (IS) studies indicated that genetic variants in CYP4F2 gene may increase the risk of IS [19–21]. CYP4F2 rs2074900 was recently reported to be related to therapeutic responses to erlotinib in sixty Han Chinese advanced non-small cell lung cancer patients received erlotinib monotherapy [14], illustrating that it may take part in the pathological process of pulmonary disease.

In the present study, we aimed to investigate the association between CYP4F2 SNPs and COPD risk in the Hainan Han population. We hope that our study may provide evidence for the role of CYP4F2 in the pathogenesis of COPD and the prevention and diagnosis of COPD in the future.

## Materials and methods

### Ethical statement

All participants were informed of the research process of the study including the procedures, results, etc., by telephone or by visit. Every participant is randomly recruited and treated fairly, and there is no priority. In this study, we only extracted the participants’ blood samples. The other data was obtained during the physical examination and did not cause too much harm to the participants, and we analyzed the related information. At present, they have signed informed consent documents. The protocols were approved by the Institutional Review Boards of the Hainan General Hospital (Med-Eth-Re [2019]42). All procedures performed in studies involving human participants were in compliance with Department of Health and Human Services (DHHS) regulations for human research subject protection.

### Study population

We collected 313 blood samples of patients who had COPD were being diagnosed at the Hainan General Hospital. The case population consisted of 238 men and 75 women, with an average age of 60.05 ± 6.478 years. All the COPD patients underwent rigorous examination, including immunohistochemical analysis and pulmonary function examination in line accordance with the criteria of the National Heart, Lung, and Blood Institute and the World Health Organization to form the Global Initiative for Chronic Obstructive Lung Disease (GOLD) [22]. The inclusion criteria: after inhalation of bronchodilator, COPD patients were forced expiratory volume (FEV1)/forced vital capacity (FVC) < 70% for the first time indicated that airflow was obstructed and could not be completely reversed. Some other causes of respiratory diseases, such as lung cancer, bronchiectasis, pulmonary fibrosis, pulmonary cystic fibrosis, diffuse bronchiolitis and bronchiolitis obliterans, were excluded.

Totally, the control group of 508 healthy people from the physical examination center of Hainan General Hospital had no history of cancer or other diseases. The control population consisted of 337 men and 171 women, with an average age of 71.80 ± 10.089 years. Moreover, the number of non-smokers was more than smokers, and there was no significant difference in the distribution of smoking status in the non-smokers and smokers (*p* = 0.082).

### SNP selection and genotyping

We randomly selected some SNPs in the CYP4F2 gene based on the dbSNP database. Each SNP had a minor allele frequency (MAF) > 5% listed in the global population of the 1000 Genome Projects (http://www.internationalgenome.org/). And then, we used the Regulome DB (http://www.regulomedb.org/) and HaploReg v4.1(https://pubs.broadinstitute.org/mammals/haploreg/haploreg.php) to predict the function of the selected variants. We extracted genomic DNA from whole blood in accordance with the instructions of the GoldMag whole blood genomic DNA purification kit (GoldMag Co. Ltd., Xi’an, China) and genomic DNA concentration was measured using the NanoDrop 2000 (Thermo Scientific, Waltham, Massachusetts, USA). Agena MassARRAY Assay Design 3.0 software was utilized to design the multiplexed SNP MassEXTEND assay and Agena MassARRAY RS1000 was used to perform SNP genotyping. Finally, we designed primers for five SNPs (rs3093203, rs3093193, rs12459936, rs3093144 and rs3093110) (Table S1) to genotype in Hainan Han populations. And we performed data sorting and analyses by Agena Bioscience TYPER 4.0 software [23].

### Statistical analysis

Student’s t-test and Pearson′s chi-square were used to assess differences in age and gender between cases and controls, respectively. And the genotype frequency of the control group was assessed as deviating from the Hardy Weinberg Equilibrium (HWE).

In addition, we used logistic regression analysis provided by the PLINK software (version 1.07) to calculate the association between SNPs and COPD risk. Haploview software (version 4.2) was applied to observe the degree of linkage between these SNPs based on a linkage disequilibrium (LD) map [15]. All *p*-values were two-tailed and p-values less than 0.01 were considered statistically significant.

## Results

### Basic information of cases and controls

The basic information of cases and controls were listed in Table S2, including age, smoking status and so on. Statistically significant difference in the distributions of gender and age between the case group and the control group were found. And the basic information of five CYP4F2 polymorphisms was displayed in Table 1. The genotype distribution of SNPs among controls were in accordance with HWE (*p* > 0.05). The frequency distribution of allele A of rs3093203 was significantly different between cases and controls (*p* = 0.005), from which we found it to be associated with an increased risk of COPD (OR = 1.40, 95%CI: 1.11–1.77) in the Hainan Han population. Also, rs3093193 G and rs3093110 G can reduce COPD risk (*p* = 0.003, *p* < 0.000) in the Hainan Han population. The Regulome DB and HaploReg v4.1 were used to predict the SNPs function shown in Table S6.

**Table 1:** Basic information and allele frequencies of the SNPs in *CYP4F2*

SNP	Chr	Gene	Alleles	Role	MAF(A)		HWE	OR(95%CI)	*p-*value
			A < B		Case	Control	*p*-value		
rs3093203	19	*CYP4F2*	A/G	3’UTR	0.292	0.227	0.704	1.40 (1.11–1.77)	**0.005**
rs3093193	19	*CYP4F2*	G/C	Intron	0.219	0.286	0.664	0.70 (0.55–0.88)	**0.003**
rs12459936	19	*CYP4F2*	T/C	Intron	0.484	0.463	0.721	1.09 (0.89–1.33)	0.398
rs3093144	19	*CYP4F2*	T/C	Intron	0.139	0.164	0.517	0.82 (0.62–1.09)	0.167
rs3093110	19	*CYP4F2*	G/A	Intron	0.059	0.123	0.838	0.45 (0.31–0.66)	***p*** **< 0.000**

95%CI: 95% confidence interval, *HWE* Hardy-Weinberg equilibrium, *MAF* minor allele frequency, *OR* odds ratio, *SNP* single-nucleotide polymorphism

*p*-value: Calculated by Pearson χ^2^ test

### Genetic model analysis between CYP4F2 variants and COPD risk

We further explored the relationship between CYP4F2 variants and COPD risk using four genetic models listed in Table 2. Individuals with rs3093203 AG-GG genotype had a much risk of COPD (OR = 1.49, 95%CI: 1.11–1.99, *p* = 0.008) compared to individuals with AA genotype in the dominant model. In the log-additive model, the results showed rs3093203 was correlated with the risk of COPD (OR = 1.41, 95%CI: 1.11–1.79, *p* = 0.004) without adjustment.

**Table 2:** Significant *CYP4F2* variants associated with COPD susceptibility

SNP	Model	Genotype	Control	Case	Unadjusted		Adjusted for Gender and Age	
					OR(95%CI)	*p*^a^-value	OR(95%CI)	*p*^b^-value
rs3093203		AA	24	22	1		1	
	Codominant	AG	182	121	1.43 (1.05–1.93)	0.023	1.15 (0.78–1.69)	0.482
		GG	300	140	1.97 (1.07–3.62)	0.031	0.99 (0.44–2.24)	0.985
	Dominant	AA	206	143	1		1	
		AG-GG	300	140	1.49 (1.11–1.99)	**0.008**	1.13 (0.78–1.63)	0.526
	Recessive	AA-AG	24	22	1		1	
		GG	482	261	1.69 (0.93–3.08)	0.084	0.94 (0.42–2.07)	0.869
	Log-additive	–	–	–	1.41 (1.11–1.79)	**0.004**	1.07 (0.80–1.46)	0.646
rs3093193		GG	39	11	1		1	
	Codominant	GC	212	115	0.74 (0.55–1.00)	0.048	0.93 (0.65–1.34)	0.704
		CC	256	187	0.39 (0.19–0.77)	**0.007**	0.49 (0.21–1.14)	0.099
	Dominant	GG	251	126	1		1	
		GC-CC	256	187	0.69 (0.52–0.91)	0.010	0.86 (0.61–1.23)	0.421
	Recessive	GG-GC	39	11	1		1	
		CC	468	302	0.44 (0.22–0.87)	0.018	0.51 (0.22–1.16)	0.108
	Log-additive	–	–	–	0.69 (0.54–0.88)	**0.002**	0.82 (0.61–1.11)	0.197
rs3093110		GG	8	0	1		1	
	Codominant	GA	109	37	0.48 (0.32–0.72)	***p*** **< 0.000**	0.46 (0.28–0.76)	**0.002**
		AA	390	275	–	–	–	–
	Dominant	GG	117	37	1		1	
		GA-AA	390	275	0.45 (0.30–0.67)	***p*** **< 0.000**	0.44 (0.27–0.72)	**0.001**
	Recessive	GG-GA	8	0	1		1	
		AA	499	312	–	–	–	–
	Log-additive	–	–	–	0.44 (0.30–0.65)	***p*** **< 0.000**	0.44 (0.27–0.72)	**0.001**

95%CI: 95% confidence interval, *OR* odds ratio, *SNP* single-nucleotide polymorphism

*p*^a^: Calculated by logistic regression analysis

*p*^b^: Calculated by logistic regression analysis adjusted for gender and age

Bold type indicates statistical significance (*p* < 0.01)

Additionally, patients with genotype CC of rs3093193 had a reduced risk of COPD in the codominant model (OR = 0.39, 95%CI: 0.19–0.77, *p* = 0.007) without adjustment. In the additive model, the SNP was associated with a reduced risk of COPD (OR = 0.69, 95%CI: 0.54–0.88, *p* = 0.002) c without adjustment for gender and age.

When compared to the GG genotype of rs3093110, heterozygous genotype GA was associated a decreased risk of COPD in the codominant model without adjustment for gender and age (OR = 0.48, 95%CI: 0.32–0.72, *p* < 0.000). Also, in the dominant model, genotype GA-AA were linked with a reduced the risk of COPD than genotype GG without adjustment (OR = 0.45, 95%CI: 0.30–0.67, *p* < 0.000). The log-additive model showed there was significantly decreased association between rs3093110 and COPD risk without adjustment for gender and age (OR = 0.44, 95%CI: 0.30–0.65, p < 0.000). After adjustment for gender and age, the variant was still related to the risk of COPD.

### Stratification analysis by gender

We also used gender stratification to investigate the association between CYP4F2 SNPs and the risk of COPD (Table 3). Pearson’s Chi-square test showed that the frequency distribution of minor allele of rs3093110 was significantly different between the male controls and the male patients (*p* = 0.000). There was a significant association between rs3093110 and COPD risk in the codominant (OR = 0.45, 95%CI: 0.27–0.73, *p* = 0.002), dominant (OR = 0.42, 95%CI: 0.26–0.69, *p* = 0.001) and log-additive (OR = 0.42, 95%CI: 0.26–0.68, *p* < 0.000) models. After adjusted for gender and age, the significant association still existed (*p* = 0.005, 0.004 and 0.004). However, there was no significant relationship between *CYP4F2* variants and COPD risk in females (Table S3).

**Table 3:** Significant *CYP4F2* variants associated with COPD susceptibility in males

SNP	Model	Genotype	Control	Case	Unadjusted		Adjusted for Gender and Age	
					OR(95%CI)	*p*^a^-value	OR(95%CI)	*p*^b^-value
rs3093203		AA	17	18	1		1	
	Codominant	AG	124	93	1.43 (1.00–2.04)	0.052	1.04 (0.64–1.68)	0.881
		GG	196	103	2.02 (1.00–4.08)	0.051	1.13 (0.42–3.03)	0.804
	Dominant	AA	141	111	1		1	
		AG-GG	196	103	1.50 (1.06–2.11)	0.022	1.05 (0.66–1.66)	0.838
	Recessive	AA-AG	17	18	1		1	
		GG	320	196	1.73 (0.87–3.43)	0.118	1.12 (0.43–2.92)	0.825
	Log-additive	–	–	–	1.42 (1.08–1.88)	0.013	1.05 (0.72–1.53)	0.799
rs3093193		GG	18	6	1		1	
	Codominant	GC	141	88	0.77 (0.54–1.08)	0.133	1.08 (0.68–1.70)	0.756
		CC	177	144	0.41 (0.16–1.06)	0.066	0.61 (0.18–2.09)	0.435
	Dominant	GG	159	94	1		1	
		GC-CC	177	144	0.73 (0.52–1.02)	0.063	1.03 (0.66–1.6)	0.907
	Recessive	GG-GC	18	6	1		1	
		CC	318	232	0.46 (0.18–1.17)	0.102	0.60 (0.18–1.99)	0.399
	Log-additive	–	–	–	0.72 (0.54–0.97)	0.030	0.96 (0.65–1.42)	0.852
rs3093110		GG	4	0	1		1	
	Codominant	GA	67	24	0.45 (0.27–0.73)	**0.002**	0.39 (0.20–0.76)	**0.005**
		AA	265	213	–	–	–	–
	Dominant	GG	71	24	1		1	
		GA-AA	265	213	0.42 (0.26–0.69)	**0.001**	0.38 (0.19–0.73)	**0.004**
	Recessive	GG-GA	4	0	1		1	
		AA	332	237	–	–	–	–
	Log-additive	–	–	–	0.42 (0.26–0.68)	***p*** **< 0.000**	0.38 (0.20–0.73)	**0.004**

95%CI: 95% confidence interval, *OR* odds ratio, *SNP* single-nucleotide polymorphism

*p*^a^: Calculated by logistic regression analysis

*p*^b^: Calculated by logistic regression analysis adjusted for gender and age

Bold type indicates statistical significance (*p* < 0.01)

### Stratification analysis by smoking status

We also used smoking status stratification to investigate the correlation between candidate SNP and COPD risk listed in Table 4. We found that rs3093110 was significantly associated with an increased risk of COPD in the non-smoker group in the codominant (OR = 0.42, 95% CI: 0.23–0.78, *p* = 0.006), dominant (OR = 0.40, 95% CI: 0.22–0.74, *p* = 0.004) and log-additive (OR = 0.40, 95% CI: 0.22–0.73, *p* = 0.003) models. But, the significant association between rs3093110 and COPD risk was not found in the smokers.

**Table 4:** Relationship of *CYP4F2* gene polymorphisms and risk of COPD stratified by Smoking status

SNP	Model	Genotype	Smoking				Non-smoking			
			Control	Case	OR(95%CI)	*p*-value	Control	Case	OR(95%CI)	*p*-value
rs3093203		AA	14	12	1		10	9	1	
	Codominant	AG	80	54	1.01 (0.52–1.96)	0.977	102	66	1.32 (0.80–2.16)	0.277
		GG	121	67	0.81 (0.22–3.02)	0.758	179	73	1.08 (0.34–3.38)	0.899
	Dominant	AA	14	12	1		10	9	1	
		AG-GG	201	121	0.98 (0.52–1.85)	0.949	281	139	1.29 (0.80–2.08)	0.301
	Recessive	AA-AG	94	66	1		112	75	1	
		GG	121	67	0.81 (0.23–2.91)	0.747	179	73	0.96 (0.31–2.94)	0.938
	Log-additive	–	–	–	0.95 (0.57–1.59)	0.855	–	–	1.19 (0.8–1.79)	0.396
rs3093193		GG	16	4	1		23	7	1	
	Codominant	GC	89	56	1.41 (0.74–2.72)	0.298	123	58	0.78 (0.49–1.25)	0.306
		CC	110	87	2.07 (0.44–9.59)	0.355	146	99	0.36 (0.13–1.02)	0.054
	Dominant	GG	16	4	1		23	7	1	
		GC-CC	199	143	1.46 (0.77–2.76)	0.241	269	157	0.71 (0.45–1.11)	0.134
	Recessive	GG-GC	105	60	1		146	65	1	
		CC	110	87	1.76 (0.39–7.90)	0.458	146	99	0.40 (0.15–1.11)	0.078
	Log-additive	–	–	–	1.42 (0.83–2.45)	0.204	–	–	0.69 (0.48–1.01)	0.054
rs3093110		GG	4	0	1		4	0	1	
	Codominant	GA	41	16	0.61 (0.24–1.51)	0.282	68	21	0.42 (0.23–0.78)	**0.006**
		AA	171	131	–	–	219	142	–	–
	Dominant	GG	4	0	1		4	0	1	
		GA-AA	212	147	0.58 (0.24–1.43)	0.237	287	163	0.40 (0.22–0.74)	**0.004**
	Recessive	GG-GA	45	16	1		72	21	1	
		AA	171	131	–	–	219	142	–	–
	Log-additive	–	–	–	0.57 (0.24–1.37)	0.211	–	–	0.40 (0.22–0.73)	**0.003**

95%CI: 95% confidence interval, *OR* odds ratio, *SNP* single-nucleotide polymorphism

*p*-value: Calculated by logistic regression analysis adjusted for gender and age

Bold type indicates statistical significance (*p* < 0.01)

#### LD and haplotype analysis

**Fig. 1:**
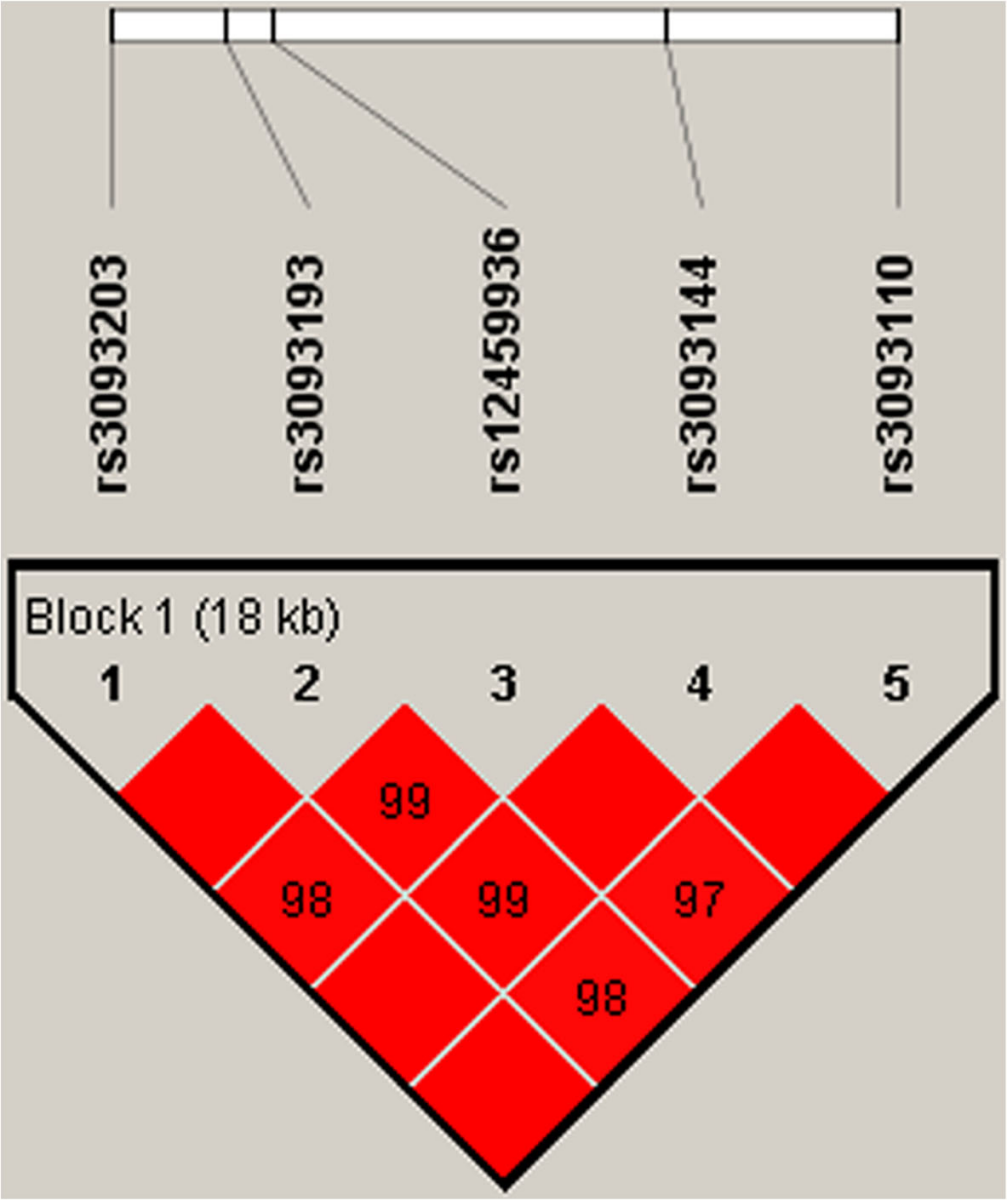
Linkage disequilibrium (LD) analysis of five SNPs in CYP4F2. The LD value is determined by r^2^ > 0.8 analyzed by Haploview software 4.2. The number in the diamonds is the LOD score of r^2^. Standard color schemes indicate the different levels of LD. Bright red: LOD > 2, D’ = 1

We also applied the Haploview software to do LD analysis in CYP4F2 variants (rs3093203, rs3093193, rs12459936, rs3093144 and rs3093110). A strong linkage mapped to a 18 kb LD block between rs3093203 and rs3093110 was found (Fig. 1). In addition, haplotypes GGCCG and GCCCA were associated with an increased risk of COPD (OR = 2.15, 95%CI: 1.46–3.16, *p* < 0.000; OR = 16.22, 95%CI: 2.19–120.30, *p* = 0.006). Whereas, haplotypes GGCCA and ACCCA decreased the risk of COPD (OR = 0.14, 95%CI: 0.04–0.49, *p* = 0.002; OR = 0.68, 95%CI: 0.54–0.86, *p* = 0.001). The relationship between haplotypes GGCTA, GCTCA and the risk of COPD were still not found (Table 5). After gender stratification, significant association between haplotypes GGCCG, ACCCA and the risk of COPD in males shown in Table S4. And when stratified analysis by smoking status (Table S5), haplotype GGCT showed the association with an increased risk of COPD (adjusted OR = 1.95, 95%CI: 1.02–3.73, *p* = 0.042) in the smokers, while haplotype ACCC significantly associated with the risk of COPD in non-smokers (OR = 1.59, 95%CI: 1.14–2.21, p = 0.006).

**Table 5:** *CYP4F2* haplotypes frequencies associated with COPD risk

Gene	SNP	Haplotype	Frequency		Unadjusted		Adjusted for Gender and Age	
			Case	Control	OR (95%CI)	*p*^a^-value	OR (95%CI)	*p*^b^-value
*CYP4F2*	rs3093203|rs3093193|rs12459936|rs3093144|rs3093110	GGCCG	0.941	0.881	2.15 (1.46–3.16)	***p*** **< 0.000**	2.21 (1.36–3.60)	**0.001**
*CYP4F2*	rs3093203|rs3093193|rs12459936|rs3093144|rs3093110	GGCTA	0.139	0.162	0.83 (0.62–1.11)	0.201	1.02 (0.72–1.46)	0.896
*CYP4F2*	rs3093203|rs3093193|rs12459936|rs3093144|rs3093110	GCTCA	0.486	0.461	1.11 (0.91–1.36)	0.317	1.21 (0.94–1.55)	0.150
*CYP4F2*	rs3093203|rs3093193|rs12459936|rs3093144|rs3093110	GGCCA	0.979	0.997	0.14 (0.04–0.49)	**0.002**	0.09 (0.02–0.41)	**0.002**
*CYP4F2*	rs3093203|rs3093193|rs12459936|rs3093144|rs3093110	ACCCA	0.706	0.776	0.68 (0.54–0.86)	**0.001**	0.91 (0.68–1.23)	0.536
*CYP4F2*	rs3093203|rs3093193|rs12459936|rs3093144|rs3093110	GCCCA	0.998	0.975	16.22 (2.19–120.30)	**0.006**	15.18 (1.65–139.60)	0.016

95%CI: 95%Confidence interval, *OR* Odds ratio, *SNP* Single nucleotide polymorphism

*p*^a^ values were calculated by logistic regression analysis without adjusted

*p*^b^ values were calculated by logistic regression analysis after adjusted for gender and age

## Discussion

In this study, we explored the connections of five variants of CYP4F2 and COPD risk in a Chinese Han population. Our results showed that rs3093203, rs3093193 and rs3093110 were significantly associated with the risk of COPD. After gender stratification, males with CYP4F2 variants (rs3093203, rs3093193 and rs3093110) showed the association with COPD risk. And the results of smoking status stratification showed that rs3093144 was associated with an increased risk of COPD in the smoker group. So, we speculated that CYP4F2 variants may be involved in the pathogenesis of COPD.

CYP4F2, located in the chromosome 19p13.12, contains 12 introns and 13 exons, which is a part of CYP4F gene cluster. Transgenic mice experiment by Lai et al. demonstrated that CYP4F2 was only expressed in the liver [24]. In the investigation of the effect of genetic variability in the CYP4F gene cluster on expression of the individual CYP4F genes in the liver, the results showed that CYP4F2 rs2108622 was correlated with an increased CYP4F2 mRNA expression level [25]. In addition, rs2108622 G was associated with IS in the Japanese men [21]. Another article reported by Liao et al. illustrated that CYP4F2 genetic variants were significantly correlated with IS risk and 20-Hydroxyeicosatetraenoic Acid Level (20-HETE) [20]. IS patients with the genotype combination of rs9333025 GG and rs2108622 GG had higher 20-HETE levels compared to IS patients with other combinations of the two variants, which demonstrated that the interaction between rs9333025 GG and rs2108622 GG can increase capability to metabolize arachidonic acid to produce 20-HETE. The evaluated 20-HETE levels were related to vascular oxidative stress, endothelial dysfunction and high peripheral vascular resistance [26, 27]. And Parker found that pharmacological inhibition of 20-HETE can abolish the myogenic response during NOS antagonism in the ovine fetal pulmonary circulation [28]. Overall, 20-HETE, a biologically active 20-carbohydrate and therapeutic intervention target, involved in a variety of vascular events, such as regulating blood pressure, renal function, cerebral blood flow and pulmonary circulation [26, 27]. So, we speculated that the polymorphisms of CYP4F2 gene affected the pathogenesis of disease by altering arachidonic acid metabolism to produce 20-HETE.

In the year of 2011, the expression of CYP4F2 was found by Falus et al. to be a rapid elevation when children with respiratory disease to polarized light therapy [29]. In 2018, rs2074900 in CYP4F2 was found to be significantly related to therapeutic responses to erlotinib in sixty Han Chinese advanced non-small cell lung cancer patients received erlotinib monotherapy [16]. The above results indicated that CYP4F2 was involved in the pathogenesis of pulmonary disease and CYP4F2 variants played a vital role in the lung disease. In our results, we did not find a link between this site and the risk of COPD, but we firstly revealed that CYP4F2 variants (rs3093203, rs3093193 and rs3093110) were associated with the risk of COPD. In future, we will increase the sample size and continue to study the results, and continue to explore the polymorphisms of CYP4F2 gene to affect the pathogenesis of COPD by changing the yield of 20-HETE.

## Conclusions

In conclusion, we revealed that rs3093203, rs3093193 and rs3093110 were significantly associated with the risk of COPD, especially in the Hainan male population. Rs3093144 may be a risk factor shown from the smoking status. The overall results may provide more evidences for COPD risk diagnosis.

## Supplementary information

**Additional file 1: Table S1.** PCR primers for amplification and extension of loci used in this study.

**Additional file 2: Table S2.** The basic information of cases and controls.

**Additional file 3: Table S3.** Significant *CYP4F2* variants associated with COPD susceptibility in females.

**Additional file 4 Table S4.***CYP4F2* haplotypes frequencies associated with COPD risk in males.

**Additional file 5: Table S5.***CYP4F2* haplotypes frequencies associated with COPD risk in smokers and non-smokers.

**Additional file 6: Table S6.** In silico analysis for SNPs function annotation.

## Data Availability

Not applicable.
