# Supplementary material for: The correlation between CYP4F2 variants and chronic obstructive pulmonary disease risk in Hainan Han population
Source: Respir Res. 2020 Apr 15;21:86. doi: 10.1186/s12931-020-01348-6 (PMC7161254; doi:10.1186/s12931-020-01348-6)
Supplement: Supplementary file 1 — Additional file 1: Table S1. PCR primers for amplification and extension of loci used in this study. [file 12931_2020_1348_MOESM1_ESM.docx]

| SNP_ID | Allele | 1st-PCR primer sequence | 2nd-PCR primer sequence | UEP sequence | Director |
| --- | --- | --- | --- | --- | --- |
| rs3093203 | A/G | ACGTTGGATGAAAGCCACCAATCCGCTATG | ACGTTGGATGGGTCACATAGTGTACTGTCC | ACATAGTGTACTGTCCTTTTATA | R |
| rs3093193 | G/C | ACGTTGGATGGTGATGAGACTAGTGATCCC | ACGTTGGATGGCCACATACACATTGATGGG | GTTTAGATAAACAGCCACA | F |
| rs12459936 | T/C | ACGTTGGATGGGTAACCATCATTCTGCTTC | ACGTTGGATGAGAGGTCGCAGTAAGCTGAG | CAGCCTGGGTGACAGAG | F |
| rs3093144 | T/C | ACGTTGGATGGGGAAGAATTGTGGCAAAGG | ACGTTGGATGAGGAGTCTCTCGTCCTTCTG | AGTTAAAAAAAAAATCCTAGATACTT | F |
| rs3093110 | G/A | ACGTTGGATGGTCTCATTGATAAGAGGGAG | ACGTTGGATGTCCTGTTATGAGGGTACAGC | CCGTCTCCCACTTCCAC | R |

Table S1. PCR primers for amplification and extension of loci used in this study

PCR: polymerase chain reaction; SNP: single-nucleotide polymorphism; UEP: unextended mini-sequencing primer.
