# Supplementary material for: The correlation between CYP4F2 variants and chronic obstructive pulmonary disease risk in Hainan Han population
Source: Respir Res. 2020 Apr 15;21:86. doi: 10.1186/s12931-020-01348-6 (PMC7161254; doi:10.1186/s12931-020-01348-6)
Supplement: Supplementary file 2 — Additional file 2: Table S2. The basic information of cases and controls. [file 12931_2020_1348_MOESM2_ESM.docx]

Table S2 The basic information of cases and controls

| Variable (s) | Case, N (%) | Control, N (%) | *p* value |
| --- | --- | --- | --- |
| Gender N (%) |  |  | 0.004^a^ |
| Male | 238 (76.0%) | 337 (66.3%) |  |
| Female | 75 (24.0%) | 171 (33.7%) |  |
| Age, year (mean ± SD) | 71.80 ± 10.089 | 60.05 ± 6.478 | < 0.001^b^ |
| Smoking status, N (%) |  |  |  |
| Smoking | 147 (47.0%) | 216 (42.5%) | 0.082^b^ |
| Non-smoking | 164 (52.4%) | 292 (57.5%) |  |
| Deletion | 2 (0.6%) |  |  |

^a^*p* were calculated by two-side chi-squared tests;

^b^*p* were calculated form samples *t* test;

*p* < 0.01 indicates statistical significance.
