# Supplementary material for: The correlation between CYP4F2 variants and chronic obstructive pulmonary disease risk in Hainan Han population
Source: Respir Res. 2020 Apr 15;21:86. doi: 10.1186/s12931-020-01348-6 (PMC7161254; doi:10.1186/s12931-020-01348-6)
Supplement: Supplementary file 3 — Additional file 3: Table S3. Significant CYP4F2 variants associated with COPD susceptibility in females. [file 12931_2020_1348_MOESM3_ESM.docx]

Table S3 Significant *CYP4F2* variants associated with COPD susceptibility in females

| SNP | Model | Genotype | Control | Case | Unadjusted | | Adjusted for Gender and Age | |
| --- | --- | --- | --- | --- | --- | --- | --- | --- |
|  |  |  |  |  | OR(95%CI) | *p*^a^-value | OR(95%CI) | *p*^b^-value |
| rs3093203 |  | AA | 7 | 4 | 1 |  | 1 |  |
|  | Codominant | AG | 58 | 28 | 1.36(0.75-2.44) | 0.308 | 1.33(0.69-2.56) | 0.390 |
|  |  | GG | 104 | 37 | 1.61(0.44-5.80) | 0.470 | 0.80(0.18-3.52) | 0.771 |
|  | Dominant | AA | 7 | 4 | 1 |  | 1 |  |
|  |  | AG-GG | 162 | 65 | 1.38(0.79-2.44) | 0.260 | 1.25(0.67-2.35) | 0.481 |
|  | Recessive | AA-AG | 65 | 32 | 1 |  | 1 |  |
|  |  | GG | 104 | 37 | 1.42(0.40-5.03) | 0.583 | 0.72(0.17-3.09) | 0.660 |
|  | Log-additive | - | - | - | 1.32(0.82-2.11) | 0.252 | 1.12(0.66-1.89) | 0.672 |
| rs3093193 |  | GG | 21 | 5 | 1 |  | 1 |  |
|  | Codominant | GC | 71 | 27 | 0.70(0.39-1.25) | 0.224 | 0.71(0.38-1.34) | 0.295 |
|  |  | CC | 79 | 43 | 0.44(0.15-1.24) | 0.121 | 0.41(0.13-1.28) | 0.126 |
|  | Dominant | GG | 21 | 5 | 1 |  | 1 |  |
|  |  | GC-CC | 150 | 70 | 0.64(0.37-1.11) | 0.109 | 0.64(0.35-1.17) | 0.146 |
|  | Recessive | GG-GC | 92 | 32 | 1 |  | 1 |  |
|  |  | CC | 79 | 43 | 0.51(0.18-1.41) | 0.194 | 0.48(0.16-1.43) | 0.187 |
|  | Log-additive | - | - | - | 0.68(0.44-1.04) | 0.073 | 0.67(0.42-1.07) | 0.091 |
| rs3093110 |  | GG | 4 | 0 | 1 |  | 1 |  |
|  | Codominant | GA | 42 | 13 | 0.62(0.31-1.25) | 0.182 | 0.59(0.28-1.24) | 0.164 |
|  |  | AA | 125 | 62 | - | - | - | - |
|  | Dominant | GG | 4 | 0 | 1 |  | 1 |  |
|  |  | GA-AA | 167 | 75 | 0.57(0.29-1.13) | 0.108 | 0.55(0.26-1.17) | 0.120 |
|  | Recessive | GG-GA | 46 | 13 | 1 |  | 1 |  |
|  |  | AA | 125 | 62 | - | - | - | - |
|  | Log-additive | - | - | - | 0.55(0.29-1.06) | 0.072 | 0.54(0.27-1.11) | 0.096 |

95%CI: 95% confidence interval; OR: odds ratio; SNP: single-nucleotide polymorphism.

*p*^a^: Calculated by logistic regression analysis.

*p*^b^: Calculated by logistic regression analysis adjusted for gender and age.

Bold type indicates statistical significance (*p* < 0.01).
