# Supplementary material for: The correlation between CYP4F2 variants and chronic obstructive pulmonary disease risk in Hainan Han population
Source: Respir Res. 2020 Apr 15;21:86. doi: 10.1186/s12931-020-01348-6 (PMC7161254; doi:10.1186/s12931-020-01348-6)
Supplement: Supplementary file 4 — Additional file 4 Table S4.CYP4F2 haplotypes frequencies associated with COPD risk in males. [file 12931_2020_1348_MOESM4_ESM.docx]

Table S4 *CYP4F2* haplotypes frequencies associated with COPD risk in males

| Gene | SNP | Haplotype | Frequency | | Unadjusted | | Adjusted for Age | |
| --- | --- | --- | --- | --- | --- | --- | --- | --- |
|  |  |  | Case | Control | OR(95%CI) | *p*-value | OR(95%CI) | *p*-value |
| *CYP4F2* | rs3093203\|rs3093193\|rs12459936\|rs3093144\|rs3093110 | GGCCG | 0.950 | 0.891 | 2.32(1.43-3.75) | **0.001** | 2.63(1.36-5.09) | **0.004** |
| *CYP4F2* | rs3093203\|rs3093193\|rs12459936\|rs3093144\|rs3093110 | GGCTA | 0.139 | 0.152 | 0.89(0.63-1.26) | 0.520 | 1.36(0.86-2.14) | 0.184 |
| *CYP4F2* | rs3093203\|rs3093193\|rs12459936\|rs3093144\|rs3093110 | GCTCA | 0.481 | 0.475 | 1.03(0.81-1.31) | 0.827 | 1.08(0.78-1.48) | 0.657 |
| *CYP4F2* | rs3093203\|rs3093193\|rs12459936\|rs3093144\|rs3093110 | GGCCA | 0.979 | 0.997 | 0.14(0.03-0.63) | 0.011 | 0.14(0.02-0.81) | 0.028 |
| *CYP4F2* | rs3093203\|rs3093193\|rs12459936\|rs3093144\|rs3093110 | ACCCA | 0.693 | 0.769 | 0.66(0.51-0.87) | **0.003** | 0.91(0.63-1.31) | 0.609 |
| *CYP4F2* | rs3093203\|rs3093193\|rs12459936\|rs3093144\|rs3093110 | GCCCA | 0.998 | 0.975 | 12.63(1.67-95.57) | 0.014 | 13.47(1.21-150.4) | 0.035 |

95%CI: 95%Confidence interval; OR: Odds ratio; SNP: Single nucleotide polymorphism.

*p*^a^ values were calculated by logistic regression analysis without adjusted.

*p*^b^ values were calculated by logistic regression analysis after adjusted for age.
