# Supplementary material for: The correlation between CYP4F2 variants and chronic obstructive pulmonary disease risk in Hainan Han population
Source: Respir Res. 2020 Apr 15;21:86. doi: 10.1186/s12931-020-01348-6 (PMC7161254; doi:10.1186/s12931-020-01348-6)
Supplement: Supplementary file 5 — Additional file 5: Table S5.CYP4F2 haplotypes frequencies associated with COPD risk in smokers and non-smokers. [file 12931_2020_1348_MOESM5_ESM.docx]

Table S5 *CYP4F2* haplotypes frequencies associated with COPD risk in smokers and non-smokers

| Gene | SNP | Haplotype | Frequency | | Unadjusted | | Adjusted for Gender and Age | |
| --- | --- | --- | --- | --- | --- | --- | --- | --- |
|  |  |  | Case | Control | OR(95%CI) | *p*-value | OR(95%CI) | *p*-value |
| Smokers |  |  |  |  |  |  |  |  |
| *CYP4F2* | rs3093203\|rs3093193\|rs12459936\|rs3093144 | GGCT | 0.140 | 0.167 | 0.80(0.53-1.23) | 0.312 | 1.95(1.02-3.73) | 0.042 |
| *CYP4F2* | rs3093203\|rs3093193\|rs12459936\|rs3093144 | GCTC | 0.483 | 0.444 | 1.17(0.87-1.57) | 0.307 | 0.90(0.58-1.41) | 0.651 |
| *CYP4F2* | rs3093203\|rs3093193\|rs12459936\|rs3093144 | GGCC | 0.078 | 0.116 | 0.65(0.39-1.09) | 0.103 | 0.73(0.33-1.59) | 0.425 |
| *CYP4F2* | rs3093203\|rs3093193\|rs12459936\|rs3093144 | ACCC | 0.296 | 0.250 | 1.26(0.90-1.76) | 0.171 | 0.98(0.59-1.62) | 0.938 |
| *CYP4F2* | rs3093203\|rs3093193\|rs12459936\|rs3093144 | GCCC | 0.003 | 0.021 | 0.16(0.02-1.26) | 0.081 | 0.11(0.01-2.49) | 0.167 |
| Non-smokers |  |  |  |  |  |  |  |  |
| *CYP4F2* | rs3093203\|rs3093193\|rs12459936\|rs3093144 | GGCT | 0.137 | 0.161 | 0.82(0.56-1.22) | 0.332 | 0.83(0.52-1.30) | 0.411 |
| *CYP4F2* | rs3093203\|rs3093193\|rs12459936\|rs3093144 | GCTC | 0.494 | 0.471 | 1.10(0.83-1.45) | 0.496 | 1.29(0.93-1.80) | 0.127 |
| *CYP4F2* | rs3093203\|rs3093193\|rs12459936\|rs3093144 | GGCC | 0.082 | 0.127 | 0.62(0.39-0.99) | 0.043 | 0.64(0.38-1.09) | 0.103 |
| *CYP4F2* | rs3093203\|rs3093193\|rs12459936\|rs3093144 | ACCC | 0.287 | 0.209 | 1.59(1.14-2.21) | **0.006** | 1.24(0.84-1.84) | 0.277 |
| *CYP4F2* | rs3093203\|rs3093193\|rs12459936\|rs3093144 | GCCC | 0.000 | 0.027 | - | - | - | - |

95%CI: 95%Confidence interval; OR: Odds ratio; SNP: Single nucleotide polymorphism.

*p*^a^ values were calculated by logistic regression analysis without adjusted.

*p*^b^ values were calculated by logistic regression analysis after adjusted for gender and age.
