# Supplementary material for: The correlation between CYP4F2 variants and chronic obstructive pulmonary disease risk in Hainan Han population
Source: Respir Res. 2020 Apr 15;21:86. doi: 10.1186/s12931-020-01348-6 (PMC7161254; doi:10.1186/s12931-020-01348-6)
Supplement: Supplementary file 6 — Additional file 6: Table S6. In silico analysis for SNPs function annotation. [file 12931_2020_1348_MOESM6_ESM.docx]

Table S6. In silico analysis for SNPs function annotation

| SNP | Chr | Gene | Allele (A<B) | RegulomeDB Score | HaploReg |
| --- | --- | --- | --- | --- | --- |
| rs3093203 | 19 | *CYP4F2* | A/G | 6 | Motifs changed, Selected eQTL hits |
| rs3093193 | 19 | *CYP4F2* | G/C | 5 | Motifs changed, Selected eQTL hits |
| rs12459936 | 19 | *CYP4F2* | T/C | No data | Motifs changed, Selected eQTL hits |
| rs3093144 | 19 | *CYP4F2* | T/C | 5 | Motifs changed, Selected eQTL hits |
| rs3093110 | 19 | *CYP4F2* | G/A | 5 | Motifs changed, Selected eQTL hits |

5 and 6 indicate that the variant has minimal binding evidence.
